# Cannabidiol as a Promising Therapeutic Option in IC/BPS: In Vitro Evaluation of Its Protective Effects against Inflammation and Oxidative Stress

**DOI:** 10.3390/ijms24055055

**Published:** 2023-03-06

**Authors:** Tadeja Kuret, Mateja Erdani Kreft, Rok Romih, Peter Veranič

**Affiliations:** Institute of Cell Biology, Faculty of Medicine, University of Ljubljana, 1000 Ljubljana, Slovenia

**Keywords:** interstitial cystitis, bladder pain syndrome, cannabidiol, urothelial cells, inflammation, oxidative stress, PPARγ/Nrf2/NFκB signaling pathways

## Abstract

Several animal studies have described the potential effect of cannabidiol (CBD) in alleviating the symptoms of interstitial cystitis/bladder pain syndrome (IC/BPS), a chronic inflammatory disease of the urinary bladder. However, the effects of CBD, its mechanism of action, and modulation of downstream signaling pathways in urothelial cells, the main effector cells in IC/BPS, have not been fully elucidated yet. Here, we investigated the effect of CBD against inflammation and oxidative stress in an in vitro model of IC/BPS comprised of TNFα-stimulated human urothelial cells SV-HUC1. Our results show that CBD treatment of urothelial cells significantly decreased TNFα-upregulated mRNA and protein expression of IL1α, IL8, CXCL1, and CXCL10, as well as attenuated NFκB phosphorylation. In addition, CBD treatment also diminished TNFα-driven cellular reactive oxygen species generation (ROS), by increasing the expression of the redox-sensitive transcription factor Nrf2, the antioxidant enzymes superoxide dismutase 1 and 2, and hem oxygenase 1. CBD-mediated effects in urothelial cells may occur by the activation of the PPARγ receptor since inhibition of PPARγ resulted in significantly diminished anti-inflammatory and antioxidant effects of CBD. Our observations provide new insights into the therapeutic potential of CBD through modulation of PPARγ/Nrf2/NFκB signaling pathways, which could be further exploited in the treatment of IC/BPS.

## 1. Introduction

Interstitial cystitis/bladder pain syndrome (IC/BPS) is a debilitating disease of the urinary bladder characterized by chronic inflammation without bacterial infection or an identifiable pathologic cause [1]. Typical clinical symptoms and signs of IC/BPS include discomfort or pain in the bladder and surrounding pelvic region associated with increased urinary frequency, urgency, and nocturia [1,2]. Although the occurrence of IC/BPS is common (the estimated prevalence is 45–300 per 100,000 women and 8–30 per 100,000 men) [3], the majority of currently available treatments focus primarily on alleviating clinical symptoms, and to date there is no therapeutic option available that would ensure long-term beneficial effects in all patients [4,5].

IC/BPS is considered a multifactorial disease with a complex pathobiology ultimately leading to chronic inflammation, bladder fibrosis and pain [6]. Several potential mechanisms for disease development have been proposed, beginning with damage and dysfunction of urothelial cells [6], highly specialized transitional epithelial cells that line the wall of most of the urinary tract [7,8]. Damage to the urothelial cell layer increases permeability, allows urinary solutes, such as urea and potassium, to enter the bladder wall, and leads to activation of an inflammatory response with increased production of various pro-inflammatory mediators [9,10,11]. A marked inflammatory response is evident in the bladders of IC/BPS patients, as infiltration with immune cells is frequently observed [12,13,14,15] and several transcriptome studies have identified an upregulated inflammatory gene signature in bladder biopsies from patients [15,16,17,18,19]. In addition, increased levels of pro-inflammatory cytokines and chemokines have been detected in urine samples from IC/BPS patients compared to controls [20,21,22,23,24,25]. Both urothelial cell damage and a sustained inflammatory response can trigger oxidative stress and excessive production of reactive oxygen species (ROS), which can in-turn stimulate and potentiate the inflammatory response, thus initiating a self-reinforcing vicious cycle that leads to chronic inflammation [26,27]. Increased ROS production is also well documented in IC/BPS, as Jiang et al. [28,29] showed increased levels of urinary oxidative stress biomarkers (8-OHdG, 8-isoprostane), whereas Ener et al. [30] found significantly lower total antioxidant capacity in serum samples from IC/BPS patients compared to controls.

For successful future treatment of patients with IC/BPS, it would be beneficial to discover and evaluate the therapeutic effects of compounds that can modulate both IC/BPS-related processes, oxidative stress, and inflammation, e.g., the cannabinoids with cannabidiol (CBD) being the main pharmacologically active phytocannabinoid most frequently used in medical treatment [31]. CBD is not psychoactive and exerts a number of beneficial pharmacological effects, including anti-inflammatory and antioxidant [32,33]. These properties, together with its low systemic toxicity, make CBD an interesting multi-target drug candidate for the treatment of IC/BPS [34]. CBD has already been shown to be effective in alleviating symptoms of IC/BPS in experimental animal models [35,36,37,38,39] and evidence suggests that CBD reduces inflammation and oxidative stress by downregulating the expression of pro-inflammatory mediators [36,40] and increasing activities of antioxidant enzymes [40] in bladder tissue. However, the exact effects of CBD specifically in urothelial cells, as well as the molecular mechanisms and downstream signaling pathways by which CBD exerts its function, remain to be elucidated.

In this study, we investigated the potential of CBD to attenuate inflammation and oxidative stress damage in tumor necrosis α (TNFα)-stimulated normal human urothelial cells SV-HUC1, which is a well described model of inflammation that mimics inflammatory changes appearing in the bladders of patients with IC/BPS [41,42,43]. We analyzed the mRNA and protein expression of various pro-inflammatory cytokines and chemokines, investigated the phosphorylation of downstream transcription factor NFκB, and determined cellular ROS generation and the expression of oxidative stress sensitive transcription factor nuclear factor erythroid 2-related factor 2 (Nrf2), as well as antioxidant enzymes, including superoxide dismutase (SOD) 1 and 2 and hem oxygenase 1 (HO1). Overall, our study provides new information about the potential effects of CBD on the attenuation of inflammation and oxidative stress and gives additional insight into the currently known mechanism of action of CBD that could be further exploited in treating IC/BPS and other related chronic inflammatory diseases.

## 2. Results

### 2.1. Unstimulated Urothelial Cells Have Higher mRNA and Protein Expression of PPARγ Compared to Other CBD-Related Receptors

**Figure 1:**
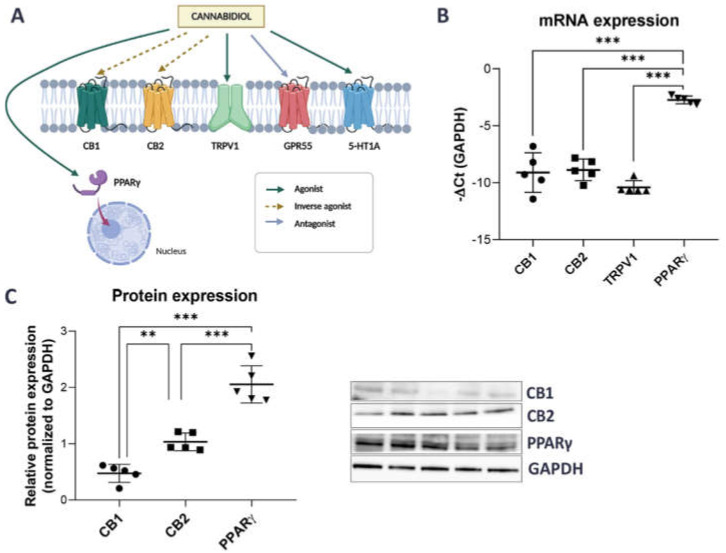
mRNA and protein expression of CBD-related receptors in SV-HUC1 cells. (**A**) CBD can mediate its effect through several receptors, including CB1, CB2, TRPV1, GPR55, 5-HT1A and nuclear receptor PPARγ [45,47]. The figure was created using Biorender.com (**B**) mRNA expression of CB1, CB2, TRPV1 and PPARγ in SV-HUC1 cells as determined by qPCR. Shown is mean ± SD negative ∆Ct of four replicates normalized to GAPDH. (**C**) Protein expression of CB1, CB2 and PPARγ receptors in SV-HUC1 cells as determined by western blots. Shown is mean relative expression ± SD of five replicates normalized to GAPDH. Representative blots for each receptor are shown. ** *p* < 0.01; *** *p* < 0.001.

Recent reports have shown that the effects of CBD are mediated not only by the binding and activation of its best known targets, the cannabinoid receptors CB1 and CB2, but also by several other CBD-related receptors, such as the nuclear peroxisome proliferator-activated receptor gamma (PPARγ), the transient receptor potential vanniloid 1 (TRPV1) channel, the G protein-coupled receptor GPR55, and the 5-HT1A serotonin receptors [44,45,46,47] (Figure 1A).

To select the most promising CBD targets for further investigation, we first searched a publicly available transcriptomic database of urothelial cells (accession number GSE2025769) and discovered that the expression of CB1, CB2, TRPV1, and PPARγ, but not GPR55 and 5-HTA1 receptors was reported [48]. Next, we therefore focused only on these four receptors (normalized counts higher than zero) and confirmed that their mRNA and protein levels (except for TRPV1) could be detected by qPCR and Western blot in SV-HUC1 cells (Figure 1B,C). mRNA and protein expression of PPARγ was significantly higher compared to the expression of other CBD-related receptors tested (Figure 1B,C).

### 2.2. Initial Optimization of Concentration of Tested Compounds and Time of Incubation

Based on our results showing higher protein expression of CB2 and PPARγ receptors compared to CB1 in urothelial cells (Figure 1C) and results from previous studies [37,38], identifying the CB2, but not CB1, receptor as a promising target to reduce symptoms of experimental cystitis and bladder inflammation in animal models, we speculated that CB2 and/or PPARγ are the receptors most probably targeted by CBD in SV-HUC1 cells.

**Figure 2:**
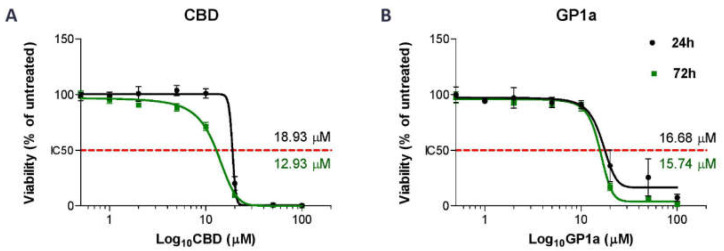
Half-maximal inhibitory dose (IC50) values of CBD- and GP1a-treated SV-HUC1 cells. SV-HUC1 were treated with increasing concentrations of CBD (**A**) or GP1a (**B**) (0, 0.5, 1, 2, 5, 10, 20, 50, 100 µM) for 24 h and 72 h and analysed with ATP viability assay. The data are normalized to the corresponding non-treated control cells (set to 100%). The mean ± SD values of three independent experiments performed in triplicates are presented. Nonlinear regression analysis of the mean cytotoxicity values of CBD and GP1a was used for IC50 determination (indicated with red dotted line).

To investigate this further, we evaluated the biological effects of GP1a, a specific CB2 receptor agonist, in addition to CBD. First, we performed a viability assay with increasing concentrations of tested compounds and incubation periods of 24 and 72 h to select the most optimal concentration of CBD and GP1a and incubation time. We confirmed that DMSO, used to prepare stock solutions of CBD and GP1a, had no significant effect on the viability of SV-HUC1 cells (Supplemental Figure S1). After 24 h of treatment, sensitivity to CBD (Figure 2A) and GP1a (Figure 2B) was similar in the SV-HUC1 cell line, with IC_50_ values of 18.93 µM and 16.68 µM, respectively. There was no statistical difference in IC_50_ value obtained in SV-HUC1 cells after 24 h or 72 h exposure to GP1a (*p* = 0.204). However, the IC_50_ value obtained after 72 h of exposure to CBD was significantly lower compared to 24 h exposure (*p* = 0.0041). Based on these results, we treated SV-HUC1 cells with concentrations of CBD and GP1a lower than their IC_50_ values for 24 h in our further experiments.

### 2.3. CBD Reduces the TNFα-Driven Release of Pro-Inflammatory Mediators from Urothelial Cells through Inhibition of NFκB Activation

Previous studies have observed anti-inflammatory effects of CBD in bladder tissues of animal models with induced IC/BPS [35]. However, how CBD affects the inflammatory response of urothelial cells has not yet been investigated. Here, we focused on measuring the most promising pro-inflammatory mediators previously reported to be significantly upregulated in an in vitro model of IC/BPS [48] and repeatedly found to be elevated in bladders [15,16,17,18,19] and urine samples from IC/BPS patients [20,21,22,23,24,25].

**Figure 3:**
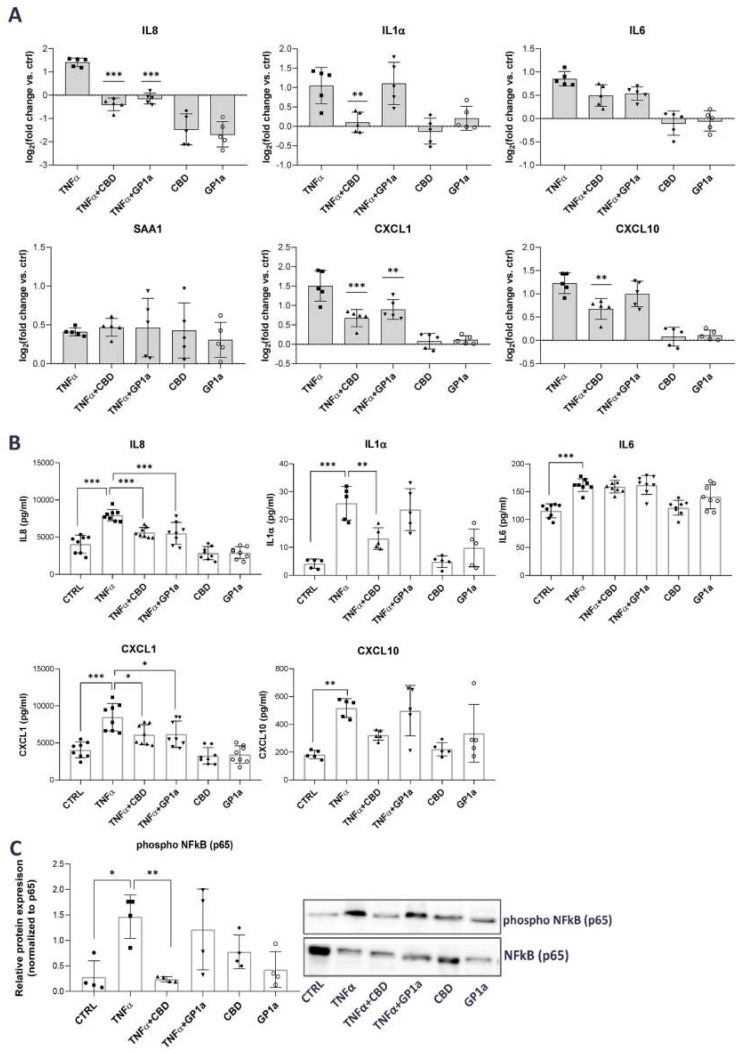
Anti-inflammatory effects of CBD in TNFα-stimulated SV-HUC1 cells. (**A**) mRNA expression of IL8, IL1α, IL6, SAA1, CXCL1 and CXCL10 in SV-HUC1 cells treated with TNFα (20 ng/mL) in the presence/absence of CBD (5 µM) or GP1a (5µM) for 24 h determined by qPCR. Shown is mean ± SD log2 fold change vs. untreated control (set to 0) determined in five independent experiments. ** *p* < 0.01, *** *p* < 0.001 vs. TNFα-treated cells. (**B**) Protein levels of IL8, IL1α, IL6, CXCL1 and CXCL10 released into the supernatants of SV-HUC1 cells treated with TNFα (20 ng/mL) in the presence/absence of CBD (5 µm) or GP1a (5 µM) for 24 h determined by ELISA. Shown is mean ± SD of concentrations determined in eight (IL8, IL6, CXCL1) or five (IL1α, CXCL10) independent experiments. (**C**) Protein levels of phosphorylated NFκB subunit p65 in SV-HUC1 cells treated with TNFα (20 ng/mL) in the presence/absence of CBD (5 µM) or GP1a (5 µM) for 24 h determined by western blot. Shown is mean ± SD relative protein expression normalized to the levels of unphosphorylated NFκB subunit p65 and representative blots of four independent experiments performed. * *p* < 0.05, ** *p* < 0.01, *** *p* < 0.001.

mRNA and protein levels of selected pro-inflammatory mediators were measured in SV-HUC1 treated simultaneously with TNFα (20 ng/mL) and CBD (5 µM) or GP1a (5 µM) for 24 h and compared with levels determined in cells treated with TNFα alone or untreated cells. Our results show that CBD was able to significantly downregulate TNFα-upregulated mRNA expression of inflammatory cytokines IL8, IL1α, and IL6, as well as chemokines CXCL1 and CXCL10, but no effect was observed on serum amyloid A1 (SAA1) mRNA expression (Figure 3A). In contrast, GP1a, a selective CB2 agonist, was able to significantly downregulate only the mRNA expression of IL8 and CXCL1 (Figure 1A). Additionally, CBD significantly decreased TNFα-driven protein release of IL8, IL1α, and CXCL1. Lower protein levels of CXCL10 were also observed after treatment with CBD and TNFα in comparison to TNFα alone, but significance was not reached. GP1a significantly attenuated the TNFα-stimulated protein release of IL8 and CXCL1 (Figure 3B). Our findings showing that simultaneous treatment with TNFα and CBD significantly attenuated IL8 release compared to TNFα alone were also confirmed using an additional urothelial cell line RT4 (Supplemental Figure S2).

To determine the potential mechanism by which CBD and GP1a may confer the observed anti-inflammatory effects, we next investigated the phosphorylation of NFκB, a known signaling pathway downstream of TNFα-stimulation [49]. In the absence of stimuli, NFκB is associated with IκBα, and therefore not activated. TNFα causes the degradation of IκBα and enables the activation of NFκB through phosphorylation [50]. In our model, we observed increased phosphorylation of the NFκB p65 subunit stimulated by TNFα, whereas CBD was able to significantly reduce this TNFα-mediated effect (Figure 3C). The relative protein expression of phosphorylated NFκB p65 subunit was normalized to the levels of unphosphorylated p65 (Figure 3C), as well as GAPDH (Supplemental Figure S3), showing the same statistical differences. No significant effect on NFκB phosphorylation was observed in the presence of GP1a (Figure 3C).

### 2.4. CBD Attenuates the TNFα-Induced ROS Formation in Urothelial Cells by Upregulating the Expression of Nrf2 and Antioxidant Enzymes

**Figure 4:**
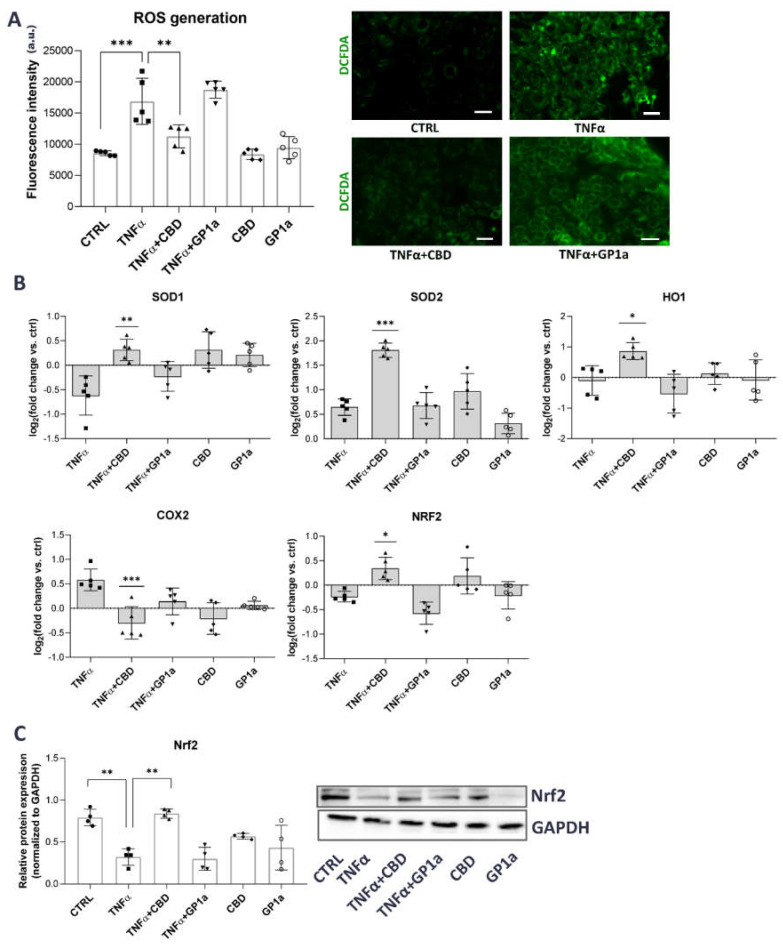
Antioxidant effects of CBD in TNFα-stimulated SV-HUC1 cells. (**A**) Cellular ROS generation in SV-HUC1 cells treated with TNFα (20 ng/mL) in the presence/absence of CBD (5 µM) or GP1a (5 µM) for 24 h and stained with DCFDA (green). Shown is mean ± SD fluorescence intensity determined in four independent experiments and representative images of three independent experiments performed. Images were taken at 10× magnification. Scale bars: 50 µm. a.u., arbitrary units, ** *p* < 0.01, *** *p* < 0.001. (**B**) mRNA expression of SOD1, SOD2, HO1, COX2 and Nrf2 in SV-HUC1 cells treated with TNFα (20 ng/mL) in the presence/absence of CBD (5 µM) or GP1a (5 µM) for 24 h determined by qPCR. Shown is mean ± SD log2 fold change vs. untreated control (set to 0) determined in five independent experiments. * *p* < 0.05, ** *p* < 0.01, *** *p* < 0.001 vs. TNFα-treated cells. (**C**) Protein levels of Nrf2 in SV-HUC1 cells treated with TNFα (20 ng/mL) in the presence/absence of CBD (5 µM) or GP1a (5 µM) for 24 h determined by western blot. Shown is mean ± SD relative protein expression normalized to the levels of GAPDH and representative blots of four independent experiments performed. ** *p* < 0.01.

Several studies suggest that inflammation leads to increased production of ROS [27] and that CBD can protect bladder tissue from oxidative stress in experimental animal models of IC/BPS by increasing the expression and activity of antioxidant enzymes [40]. Hence, we were interested to determine whether this is also true in human urothelial cells in vitro. TNFα stimulation increased ROS production in SV-HUC1 cells, as determined by the DCFDA assay kit and fluorescence microscopy, compared to untreated cells, and this could be attenuated by concomitant treatment with CBD, but not with GP1a (Figure 4A).

We next focused on possible molecular mechanisms that could be responsible for this CBD-mediated effect. Nrf2 is a key cellular oxidative stress sensor that can induce transcription of antioxidant genes and thus protect cells from oxidative stress [51]. Our results show that TNFα stimulation decreased the expression of Nrf2 and SOD1 but increased the expression of COX2 compared to untreated cells. Simultaneous treatment with CBD and TNFα increased the expression of Nrf2 and the antioxidant genes SOD1, SOD2, and HO1, as well as decreased the expression of COX2 compared to TNFα-treated cells (Figure 4B,C). No significant effects of TNFα or CBD were observed for the mRNA expression of other oxidative stress-related genes, including NQO1 and KEAP1 (Supplemental Figure S4).

### 2.5. CBD Effects in Urothelial Cells Are Mediated through PPARγ Receptor Activation

**Figure 5:**
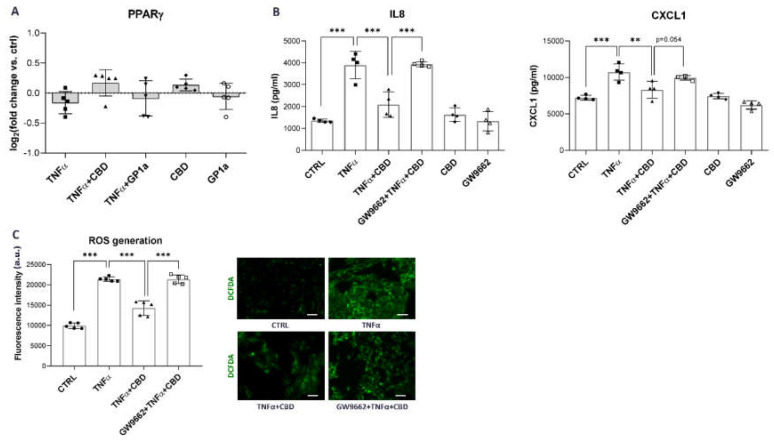
Diminished anti-inflammatory and anti-oxidant effects of CBD in the presence of PPARγ inhibitor. (**A**) mRNA expression of PPARγ in SV-HUC1 cells treated with TNFα (20 ng/mL) in the presence/absence of CBD (5 µM) or GP1a (5 µM) for 24 h determined by qPCR. Shown is mean ± SD log2 fold change vs. untreated control (set to 0) determined in five independent experiments. (**B**) Protein levels of IL8, and CXCL1 released into the supernatants of SV-HUC1 cells treated with TNFα (20 ng/mL) in the presence/absence of CBD (5 µm), with/without a 2 h pre-incubation with a selective PPARγ antagonist GW9662, for 24 h determined by ELISA. Shown is mean ± SD of concentrations determined in four independent experiments. (**C**) Cellular ROS generation in SV-HUC1 cells treated with TNFα (20 ng/mL) in the presence/absence of CBD (5 µM) with/without a 2 h pre-incubation with a selective PPARγ antagonist GW9662 for 24 h and stained with DCFDA (green). Shown is mean ± SD fluorescence intensity determined in four independent experiments and representative images of three independent experiments performed. Images were taken at 10× magnification. Scale bars: 50 µm. a.u., arbitrary units; ** *p* < 0.01; *** *p* < 0.001.

Since our results consistently showed that CBD is capable of eliciting more prominent anti-inflammatory and antioxidant effects compared to GP1a, a selective CB2 receptor agonist, we speculated that the PPARγ receptor, which is one of the CBD-related receptors most highly expressed in SV-HUC1 cells (Figure 1B,C), might be involved in the observed effects. We first determined the expression of PPARγ in SV-HUC1 cells, treated with TNFα and/or TNFα in combination with CBD or GP1a and/or CBD and GP1a alone. Our qPCR analysis showed a slight, although not significant, upregulation of PPARγ mRNA expression in SV-HUC1 cells in the presence of CBD, whereas no difference was observed when cells were exposed to TNFα or GP1a compared to untreated cells (Figure 5A).

To determine whether the observed CBD effects were mediated by PPARγ, we treated SV-HUC1 cells with GW9662, a selective PPARγ antagonist, for 2 h prior to treatment with CBD and TNFα. The CBD-mediated anti-inflammatory effects were almost completely abolished when the PPARγ receptor was inhibited, resulting in significantly increased protein release of IL8 and CXCL1 (Figure 5B) compared to CBD treatment without PPARγ inhibition. In contrast, no effect of GW9662 on protein levels of IL8 and CXCL1 was observed when cells were treated with GP1a (Supplemental Figure S5), indicating a specific inhibitory effect on PPARγ. Moreover, pretreatment with the PPARγ antagonist reversed the antioxidant effect of CBD, resulting in the increased formation of ROS (Figure 5D) compared to CBD treatment without GW9662 preincubation. Overall, our results show that CBD suppresses pro-inflammatory and oxidative stress-related signaling in TNFα-stimulated urothelial cells through activation of the PPARγ receptor, which has been shown to directly interact with the p65 subunit of NFκB [52,53] and regulate Nrf2 transcription factor expression [53,54].

## 3. Discussion

Pharmacologically active plant compounds, including CBD, show promising but underexplored potential in the prevention and treatment of chronic inflammatory diseases. CBD is nonpsychotropic and, due to its anti-inflammatory and antioxidant properties, may represent a prototype for future drug development for those human diseases in which both persistent inflammation and oxidative stress play key roles in their development and progression [55]. One of these diseases is IC/BPS, a multifactorial inflammatory disease of the urinary bladder, for which no safe, and effective therapeutic approach currently exists [56]. Several studies have already investigated the use of CBD in experimental animal models of IC/BPS, demonstrating a significant reduction in pain severity and inflammation, increased activity of the antioxidant defense mechanism, and reduced bladder damage [37,38,39,40]. However, very few data are available on the protective effects and mechanism of action in urothelial cells, the main effector cells in IC/BPS pathobiology [57].

CBD exerts its pharmacological function by binding and activating its receptors. The most well-described are the cannabinoid receptors CB1 and CB2 [58]. However, CBD has also been shown to act on a variety of other receptors, including but not limited to PPARγ [59] and TRPV1 [60]. The expression of functional CB1 and CB2 receptors has been demonstrated in normal human [61,62], rat, and mouse bladder urothelium [62], as well as in the normal human urothelial cell lines HCV29 and UROtsa [63] and the transformed RT4 cell line [64], whereas no reports to date exist regarding the expression of CBD-related receptors in normal urothelial cells SV-HUC1, which are often used as an in vitro model of IC/BPS [35,41,42,43]. We show that SV-HUC1 cells express CB1, CB2, and PPARγ receptors, but PPARγ expression was significantly higher compared to CB1 and CB2 expression (Figure 1). PPARγ is expressed in the healthy human urothelium, especially in the superficial layer [65] and plays a critical role in inducing terminal differentiation of urothelial cells, by upregulating cytokeratins CK13 and CK20, tight junction-associated claudin 3, and uroplakins UPK1a and UPK2 [66,67,68]. Since terminally differentiated urothelial cells are absent or present only in limited numbers in the bladders of IC/BPS patients due to urothelial denudation [57] and PPARγ is also importantly involved in regulation of inflammatory response to urinary tract infection [69], we suggest that it might play an important, but not yet fully investigated, role in sustained inflammation, characteristic for IC/BPS. Based on results from other studies, showing that CB2, but not CB1, activation is involved in attenuation of bladder inflammation and the severity of experimental cystitis [37,38], we speculated that CB2 and PPARγ are the most probable targets of CBD in SV-HUC1 cells. Therefore, we examined the biological effects of GP1a, a selective CB2 receptor agonist, in addition to CBD. The purpose of including GP1a was to determine whether CBD primarily binds and activates the CB2 or PPARγ receptor in urothelial cells.

We discovered that both tested compounds were able to modulate inflammation in TNFα-stimulated SV-HUC1 cells, with significant attenuation of protein release of IL1α, IL8, and CXCL1, after CBD treatment. These results confirm a previous study on IC/BPS, which showed decreased expression of IL1α and IL8 in mouse bladders after CB2 receptor activation [36]. The suppressive in vitro inhibitory effects of CBD on the production and release of IL1, IL8, and CXCL1 have also been previously shown in other pre-clinical in vitro inflammatory models using various cell types, including monocytes [70], macrophages [71], epithelial cells, and fibroblasts [72,73], stimulated with LPS or TNFα. A previous study showed that CBD has a similar anti-inflammatory effect to dexamethasone in macrophages by attenuating the LPS-induced production of NO, IL6, and TNFα through inhibition of NFκB p65 phosphorylation [74]. Since the molecular mechanism underlying the observed anti-inflammatory effects of CBD in urothelial cells has never been studied before, we next investigated the activation of the transcription factor NFκB, which plays a central role in chronic inflammatory diseases characterized mainly by an inflammatory and innate immune response, including IC/BPS [75,76,77,78]. We show that CBD suppresses the phosphorylation of the p65 subunit of NFκB, promoted by TNFα stimulation, (Figure 3). Our results are in line with previous studies that identified NFκB as a key transcription factor affected by CBD, particularly in neuroinflammatory diseases [79,80]. Similarly, CBD inhibited NFκB phosphorylation in UV-irradiated skin keratinocytes [81], LPS-stimulated RAW 264.7 macrophages [74], as well as in mouse models of endometriosis [82] and alcoholic fatty liver disease [83]. Overall, our data suggest that CBD has potent immunosuppressive effects on key transcription factors and inflammatory mediators that are major constituents and perpetuators of the immune response in IC/BPS.

Given that the production of ROS is central to the progression of many inflammatory diseases [27] and has already been identified as an important underlying feature in IC/BPS pathobiology [28,84,85], we herein evaluated the effects of TNFα on the production of ROS and how this is affected by concomitant treatment with CBD. The production and maintenance of controlled levels of intracellular ROS are critical for cells to perform a number of physiological functions, including maintenance of redox homeostasis, cell cycle signaling, and hormone production [86]. When this homeostasis is disrupted, either by the overproduction of ROS or an inefficient ROS scavenging system, it leads to oxidative stress and eventually cell death and tissue destruction [87]. First, our study confirmed that TNFα stimulates the production of ROS (Figure 4). This is a well-documented effect that has been observed in various TNFα-stimulated cell types, including endothelial cells [88,89], epithelial cells [90,91,92], hepatic cells [93], fibroblasts [94], and cardiac myocytes [95]. On the other hand, we show that CBD was able to significantly attenuate these TNFα-mediated effects (Figure 4). CBD has previously been shown to have considerable antioxidant effects in a variety of tissue types and cell models, such as keratinocytes [81,96], endothelium [97,98], and microglia [99], mainly by increasing the expression and activity of the redox-sensitive transcription factor Nrf2, which was also shown in our study (Figure 4). On the other hand, pro-oxidant capacity of CBD has also been reported, depending on the cell model, concentration of CBD and time of incubation [87]. For example, CBD was shown to increase ROS production in human monocytes [100], breast cancer cells [101], and glioma cells [102]. In the present study, the possible differences in anti-inflammatory and antioxidant effects of CBD that would depend on its dose or time of incubation or CBD were not evaluated. This should be studied in the future, implementing different in vitro models, using various urothelial cell lines or animal models with experimentally induced IC/BPS in order to draw more prominent conclusions and justify the use of CBD in a clinical trial for patients with IC/BPS.

Nrf2 was recently identified as one of the contributors to IC/BPS by Ni et al. [85]. They showed that *nrf2* knockout mice with an experimentally induced cystitis developed more severe symptoms of IC/BPS and bladder injury with structural destruction of the urothelium compared to wild type mice [85]. In addition, D’Amico et al. [94] have shown that the use of bioactive olive oil compounds to treat mice with induced cystitis reduces bladder damage and oxidative stress by upregulating the Nrf2/HO1 pathway, restoring levels of antioxidant enzymes, and reducing lipid myeloperoxidation in the bladders [103]. Nrf2 binds to antioxidant response elements (AREs) to orchestrate the expression of antioxidant enzymes, including SOD and HO1, to promote a reduction in ROS [51]. In addition to Nrf2, CBD has also been shown to increase the expression of SOD and HO1 in keratinocytes [96], adipose tissue-derived mesenchymal stem cells [104], neuroblastoma cells [105], and smooth muscle cells [106]. A previous study not only showed increased expression and activity of phosphorylated Nrf2, but also increased expression and activity of various antioxidant enzymes, including SOD, following CBD treatment in the keratinocytes of control rats as well as in keratinocytes from skin exposed to both UVA and UVB radiation [80]. All of this is also evident in our study, in which CBD increased mRNA levels of the antioxidant genes SOD1, SOD2, and HO1 in urothelial cells (Figure 4), presumably through activation of Nrf2. However, to confirm our findings, protein levels as well as the activity of antioxidant enzymes should be measured, along with analysis of phosphorylation and nuclear translocation of Nrf2 and/or using Nrf2 knockdown cell lines. Numerous data suggest that HO1 has diverse antioxidant and anti-inflammatory abilities, making HO1 inducers such as CBD promising therapeutic agents [107]. Interestingly, Nrf2 and NFκB pathways co-regulate cellular responses to oxidative stress and inflammation [108]. Pharmacological and genetic studies suggest that there is a functional interaction between these two important signaling pathways. The absence of Nrf2 can enhance NFκB activity, leading to increased cytokine production, whereas NFκB can modulate Nrf2 transcription and activity, with both positive and negative effects on target gene expression. The Nrf2-NFκB crosstalk enables fine-tuning of dynamic responses to different environmental stimuli [109,110]. Therefore, it could be suggested that CBD-mediated alterations in Nrf2/NFκB pathways are one of the key points in modulating intracellular redox homeostasis and determining cellular response under oxidative stress and associated chronic inflammation [111].

Finally, we show that the effects of CBD on human urothelial cells are probably not, or are only slightly CB2 receptor-dependent, because the effects of GP1a, a selective CB2 agonist, were not as apparent as the effects of CBD. Instead, PPARγ receptor was highly expressed in unstimulated, as well as TNFα-stimulated SV-HUC1 cells with slight upregulation in the presence of CBD. We proved that inhibition of PPARγ resulted in reduced anti-inflammatory and antioxidant effects of CBD (Figure 5). CBD is an agonist of PPARγ [53], which has been shown to regulate NFκB signaling, either by binding directly to NFκB, which prevents its interaction with promoter regions of target genes, or alternately, by binding to the promoter region of NFκB target genes to prevent their activation [32]. While the direct mechanism by which PPARγ controls NFκB in the urothelium is unclear, current evidence suggest that PPARγ is involved in the modulation of inflammation by regulating the expression of NKκB p65 subunit after urinary tract infection, which leads to inhibition of pro-inflammatory gene expression such as COX2, IL1, and IL8 [52,69], which was also shown in this study. In addition, PPARγ also cooperates with Nrf2 by binding to the specific elements in the promoter region of Nrf2 as well as the genes it regulates, including HO1 and SOD [32,112,113]. In addition, CBD can stimulate the production and activity of the endocannabinoids anandamide and 2-arachidonoyl-glycerol, which are also PPARγ agonists, further contributing to the attenuation of inflammation and ROS generation [114].

Our study has its limitations. First, the majority of our conclusions were drawn after obtaining results from only one urothelial cell line (SV-HUC1), and a second cell line should be employed for confirmation in the future studies. Second, to validate our findings regarding the roles of the PPARγ receptor and the Nrf2 transcription factor in CBD-mediated effects, future studies should implement a PPARγ/Nrf2 knockdown cell line or a PPARγ/Nrf2 knockdown mouse model with induced IC/BPS. Third, the expression of antioxidant enzymes (SOD1, SOD2, and HO1) was only determined on mRNA but not on protein levels. To confirm that the effects of CBD are indeed mediated through increased activity of the antioxidant defense mechanism, the protein levels as well as enzymatic activity should be determined in future studies. In addition, we determined the activation of NFkB only by measuring the protein levels of the phosphorylated form of the p65 subunit. However, to confirm its activation, its translocation to the nucleus should also be determined.

To the best of our knowledge, our study provides the first in vitro characterization of CBD-mediated anti-inflammatory and antioxidant effects in human urothelial cells after inflammatory challenge with TNFα and gives additional insight into the currently known mechanism of action of CBD. CBD might exhibit anti-inflammatory and antioxidant effects by either directly or indirectly modulating the PPARγ-NFκB-Nrf2 signaling axes in urothelial cells, which may be important for breaking the vicious and self-reinforcing cycle of oxidative stress and inflammation in IC/BPS. Given the great interest in the identification of natural compounds for the prevention and/or progression of inflammatory diseases, the results of the present study may offer novel perspectives for development of an optimal therapeutic approach in IC/BPS and other chronic inflammatory diseases.

## 4. Materials and Methods

### 4.1. Cell Culture

Human normal urothelial cells SV-HUC1 (CRL-9520, ATCC, Manassas, VA, USA) were grown in 75 cm^2^ cell culture flasks in basal media consisting of equal parts of advanced Dulbecco’s modified Eagle’s medium (A-DMEM) (Gibco, Thermo Fisher Scientific, Waltham, MA, USA) and F12 (HAM) (Sigma Aldrich, St. Louis, MO, USA), supplemented with 5% fetal bovine serum (FBS) and 4 mM GlutaMAX (both Gibco, Thermo Fisher Scientific, Waltham, MA, USA). Cells repeatedly tested negative for mycoplasma infection using MycoAlert mycoplasma detection kit (Lonza, Basel, Switzerland). For the experiments, SV-HUC1 cells were seeded into appropriate plates/chambers at a seeding density of 3 × 10^4^ cells/cm^2^ and grown until reaching 70–80% confluency (approximately 3–4 days) before performing experiments. All experiments were performed in serum-free basal media.

### 4.2. Materials

Human recombinant TNFα was purchased from Cayman Chemicals, USA, reconstituted in sterile PBS to a stock concentration of 25 mg/mL, aliquoted and stored at −80 °C. Working concentration of 20 ng/mL was prepared in serum-free cell culture media. CBD, CB2 receptor agonist GP1a, and selective PPARγ antagonist GW966 were purchased from Tocris, Bio-Techne Ltd., Abingdon, UK, reconstituted in DMSO to a stock concentration of 25 mM (CBD and GP1a) or 10 mM (GW9662), aliquoted and stored at −20 °C (CBD) or room temperature (RT; GP1a, GW9662). Working concentrations were prepared in serum-free culture media. Final concentrations used in experiments were 5 µM for CBD and GP1a and 20 µM for GW9662.

### 4.3. Cell Experiments

To mimic a proinflammatory environment, cells were treated with 20 ng/mL human recombinant TNFα (Cayman Chemicals, Ann Arbor, MI, USA) for 24 h in serum-free basal media, as previously described [48]. Untreated cells grown in serum-free basal media served as controls. To evaluate the effect of CBD and GP1a, cells were treated simultaneously with TNFα (20 ng/mL) and CBD (5 µM) or GP1a (5 µM) or with CBD (5 µM) or GP1a (5 µM) alone for 24 h in serum free media, unless otherwise stated. To assess the role of PPARγ receptor in CBD-mediated effects, cells were pre-treated with PPARγ inhibitor GW9662 (20 µM) for 2 h followed by the addition of TNFα (20 ng/mL) in combination with CBD (5 µM) for another 24 h. To evaluate the effect of DMSO in which stock solutions of CBD, GP1a, and GW9662 were prepared, cells were treated with 0.02% DMSO, corresponding to the working concentration used in the experiments, in the presence/absence of TNFα for 24 h.

### 4.4. Viability Assay

For viability assays, SV-HUC1 were seeded in 96-well plates and grown until reaching 70–80% confluency. Subsequently, cells were treated with increasing concentrations of CBD or GP1a (0.5–100 µM) or DMSO (0.002–0.4%) in serum-free basal media for 24 h and 72 h with fresh cell media replacement every 24 h. Cell viability was determined using CellTiter-Glo^®^ Luminescent Cell Viability Assay (Promega, Madison, WI, USA) following manufacturer’s instructions. Luminescent signal proportional to the amount of ATP present was subsequently measured using a microplate reader (Safire; Tecan, Mannedorf, Switzerland). A viability assay was performed in triplicate in three independent experiments. The results were expressed as percentage of luminescence signal intensity of untreated controls (set to 100).

### 4.5. RNA Isolation, Reverse Transcription and qPCR

Total RNA was isolated from SV-HUC1, grown on 24-well plates, treated with/without TNFα, CBD, or GP1a for 24 h, using Quick-RNA Microprep Kit (Zymo Research, Irvine, CA, USA), according to manufacturer’s instructions with on column genomic DNA digestion. The concentration and purity of isolated RNA were assessed with a Qubit RNA Broad Range Assay Kit on Qubit Flex Fluorimeter (both Invitrogen, Thermo Fisher Scientific, Waltham, MA, USA) and NanoDropTM 1000 (Thermo Fisher Scientific), respectively. Reverse transcription of 1 μg of total RNA/sample was performed with Promega Reverse Transcription System Kit (Promega, Madison, WI, USA) following manufacturer’s instructions. qPCR analysis was performed in triplicates on LightCycler^®^ 480 PCR System in LightCycler^®^ 480 Multiwell Plates 384 (both Roche, Basel, Switzerland), using self-designed primers (Integrated DNA Technologies, Coralville, IA, USA) and 5× HOT FIREPol EvaGreen qPCR Mix Plus (Solis BioDyne, Tartu, Estonia). Sequences of primers used for qPCR are listed in Supplemental Table S1. Expression of GAPDH was used as endogenous control to normalize the data. Data were analyzed with the comparative 2^−∆∆Ct^ method and presented as log_2_fold change of TNFα-, CBD-, and GP1a-treated cells vs. untreated controls (set to 0). The results showing differences in CBD-related receptor expression in SV-HUC1 cells were analyzed with the comparative Ct method relative to the expression of endogenous control (GAPDH) and presented as negative ∆Ct between the Ct of gene of interest and the Ct of endogenous control.

### 4.6. Enzyme-Linked Immunoassays

The supernatants of SV-HUC1 cells, seeded in 12- or 24-well plates and treated with/without TNFα, CBD, GP1a and/or PPARγ receptor inhibitor GW9662 for 24 h, were collected, centrifuged (200× *g*, 5 min, RT) and stored at −80 °C until analysis. Enzyme-linked immunoassays (ELISA) were performed using commercial ELISA kits in duplicates, according to manufacturer’s instructions. Absorbance at 450 nm with a reference wavelength set at 570 nm was measured on a microplate reader (Safire; Tecan, Mannedorf, Switzerland). The following ELISA kits were used in the present study: human IL8 ELISA MAX™ Deluxe Set, human IL1α MAX™ Deluxe Set, human CXCL10 ELISA MAX™ Deluxe Set (all Bio Legend, San Diego, CA, USA), human CXCL1 Duo Set Kit (R&D Systems, Minneapolis, MN, USA), and human IL6 ELISA Kit (Cayman Chemicals, Ann Arbor, MI, USA).

### 4.7. Western Blots

For Western blots, SV-HUC1 cells were seeded in 12-well plates and treated with/without TNFα, CBD, and GP1a for 24 h. After the treatment, the cells were collected and lysed in ice-cold RIPA lysis buffer (Merck, Kenilworth, NJ, USA), containing a Halt™ Protease and Phosphatase Inhibitor Cocktail (Thermo Fisher Scientific, Waltham, MA, USA). Total protein levels were quantified using the Pierce BCA Protein Assay Kit (Thermo Fisher Scientific, USA). Equivalent concentrations of protein (25 µg/lane) were separated using 4–20% Novex WedgeWell Tris-Glycine Gels (Invitrogen, Carlsbad, CA, USA) and then transferred onto a nitrocellulose membrane (Sigma-Aldrich, St Louis, MO, USA). The membranes were blocked in blocking buffer consisting of 5% skim milk or 5% BSA in 0.1% Tris Buffered saline/Tween 20 (TBS-T) for 2 h at RT and incubated overnight at 4 °C with primary antibodies against CB1 receptor (diluted 1:200 in 5% BSA/TBS-T; 101500, Cayman Chemicals, Ann Arbor, MI, USA), CB2 receptor (diluted 1:500 in 5% BSA/TBS-T, 101550, Cayman Chemicals, USA), PPARγ (diluted 1:500 in 5% skim milk/TBS-T; sc7273, Santa Cruz, Dallas, TX, USA), TRPV1 (diluted 1:200 in 5% 5% BSA/TBS-T; orb13755, Biorybt, Cambridge, UK), Nrf2 (diluted 1:500 in 5% skim milk/TBS-T; D1Z9C, Cell Signaling, Danvers, MA, USA), NFκB p65 (diluted 1:1000 in 5% BSA/TBS-T; D14E12, Cell Signaling, Danvers, MA, USA), phospho NFκB p65 (diluted 1:1000 in 5% BSA/TBS-T; 93H1, Cell Signaling, Danvers, MA, USA), and GAPDH (diluted 1:1000 in 5% BSA/TBS-T; sc47724, Santa Cruz, Dallas, TX, USA). The next day, the membranes were washed with TBS-T and immediately incubated for 1 h at RT with mouse or rabbit secondary antibodies conjugated with horseradish peroxidase (diluted 1:1000 in 5% BSA/TBS-T, A4412 and A6154, Sigma-Aldrich, USA). Visualization of the protein bands was performed using the SuperSignal West Pico or Femto Chemiluminescent Substrate (Thermo Fisher Scientific, USA), and the iBright FL1500 imaging system (Thermo Fisher Scientific, USA). iBright Firmware 1.7. (Thermo Fisher Scientific, USA) was used to perform the densitometric analysis, normalized to the expression of GAPDH, used as loading control, unless otherwise stated.

### 4.8. Cellular ROS Detection

The ROS formation in SV-HUC1 cells was detected by utilizing the cell-permeable reagent 2′,7′-dichlorofluorescein (DCFDA), which is oxidized by ROS to form a fluorescent compound (ab113851; Abcam, Cambridge, UK), according to the manufacturer’s instructions. For quantification, 50,000 cells/well were seeded in 96-well plates and grown for 24 h. Subsequently, cells were treated with/without TNFα and/or CBD, GP1a, or PPARγ inhibitor GW9662 for 24 h, washed, incubated with 25 µM of DCFDA solution for 45 min at 37 °C in the dark, and rinsed with the dilution buffer. Fluorescence was measured at excitation wavelength of 485 nm end emission wavelength of 529 nm on a microplate reader (Safire; Tecan, Mannedorf, Switzerland). For immunofluorescence microscopy, cells were seeded in chamber slides with a removable 8 well chamber (Ibidi, Fitchburg; Dane County, WI, USA) at a seeding density of 3 × 10^4^/cm^2^, grown until reaching 70–80% confluency, and treated with/without TNFα and/or CBD, GP1a, or PPARγ inhibitor GW9662 for 24 h. After treatment, cells were incubated with 25 µM of DCFDA solution for 45 min at 37 °C in the dark, and washed with the dilution buffer. The samples were imaged with a fluorescence microscope AxioImager.Z1 equipped with ApoTome (Carl Zeiss MicroImaging GmbH, München, Germany).

### 4.9. Statistical Analysis

Statistical analysis was performed using Graph Pad Prism software 8.01 (Graphpad Software Inc., San Diego, CA, USA). The normality of data distribution was investigated by the Shapiro–Wilk test. Due to the normal distribution of the data, summary statistics are expressed as means and standard deviations (SD) unless otherwise stated. Multiple group comparisons were performed by analysis of variance (normal distribution) test with adjustments for multiple comparisons using Dunn’s post hoc test. Nonlinear regression analysis of the mean cytotoxicity values for CBD and GP1a was used for IC_50_ determination. All tests were two-tailed and *p* values of <0.05 were regarded as statistically significant.

## Data Availability

The original contributions presented in the study are included in the article/supplementary material, further inquiries can be directed to the corresponding author.

## Supplementary Materials

Supplementary materials can be found at https://www.mdpi.com/article/10.3390/ijms24055055/s1.

## Abbreviations

IC	Interstitial cystitis
BPS	Bladder pain syndrome
ROS	Reactive oxygen species
CBD	Cannabidiol
qPCR	Quantitative polymerase chain reaction
Nrf2	Nuclear factor erythroid 2-related factor 2
SOD	Superoxide dismutase
HO1	Hem oxygenase 1
COX2	Cyclooxygenase 2
IL	Interleukin
CXCL	(C-X-C motif) ligand
SAA	Serum amyloid A
Ct	Threshold cycle
