# Supplementary material for: Cannabidiol as a Promising Therapeutic Option in IC/BPS: In Vitro Evaluation of Its Protective Effects against Inflammation and Oxidative Stress
Source: Int J Mol Sci. 2023 Mar 6;24(5):5055. doi: 10.3390/ijms24055055 (PMC10003465; doi:10.3390/ijms24055055)
Supplement: Supplementary file 1 [file ijms-24-05055-s001.zip › Suppl_Table_S1.pdf]

Supplementary Table S1. List of mRNA primers used for qPCR.

mRNA	Forward sequence (5'-3')	Reverse sequence (5'-3')
GAPDH	CGGATTTGGTCGTATTGG	TCCTGGAAGATGGTGATG
IL1α	AGATGCCTGAGATACCCAAAACC	CCAAGCACACCCAGTAGTCT
IL6	CCCTGAGAAAGGAGACATGTA	CCTCTTTGCTGCTTTCACACA
IL8	TTTTGCCAAGGAGTGCTAAAGA	AACCCTCTGCACCCAGTTTTC
CXCL1	AACCGAAGTCATAGCCACACT	TCTGGTCAGTTGGATTTGTCACT
CXCL10	AGTGGCATTCAAGGAGTACCT	GATCTCAACACGTGGACAAAATTG
SAA1	AGAGATTCTTTGGCCATGGTG	TCGGAAGTGATTGGGGTCTT
NRF2	CATTCTGCTGAGTTTGATTGGGG	TTGTGGAACTGGGTCTGAGTAT
SOD1	ACAAACATCCTCTGCCCGAT	AACGACTTCCACCGTTTCCT
SOD2	TGCTCCCACACATCAATCC	GGTTGGCTTGGTTTCAATAAGG
HO1	AAGACTGCGTTCCTGCTCAAC	AAAGCCCTACAGCAACTGTCG
COX2	AAGCGAGGGCCAGCTTTCACCA	CCAAAGACCTCCTGCCCCACAGC
KEAP1	GTGTCCATTGAGGGTATCCACC	GCTCAGCGAAGTTGGCGAT
NQO1	GAAGAGCACTGATCGTACTGGC	GGATACTGAAAGTTCGCAGGG
CB1	GATACCACCTTCCGCACCAT	TACCCTAATTTGGATGCCATGTC
CB2	ATCATGTGGGTCCTCTCAG	GATTCCGGAAAAGAGGAAGG
TRPV1	GCCTGAAGGAGCTTGTCAAC	CGCCCTTTGGTTTTCTTAAA
PPARγ	TACTGTCGGTTTCAGAAATGCC	GTCAGCGGACTCTGGATTCAG
