# Supplementary figures and images for: Cannabidiol as a Promising Therapeutic Option in IC/BPS: In Vitro Evaluation of Its Protective Effects against Inflammation and Oxidative Stress
Source: Int J Mol Sci. 2023 Mar 6;24(5):5055. doi: 10.3390/ijms24055055 (PMC10003465; doi:10.3390/ijms24055055)

# DMSO

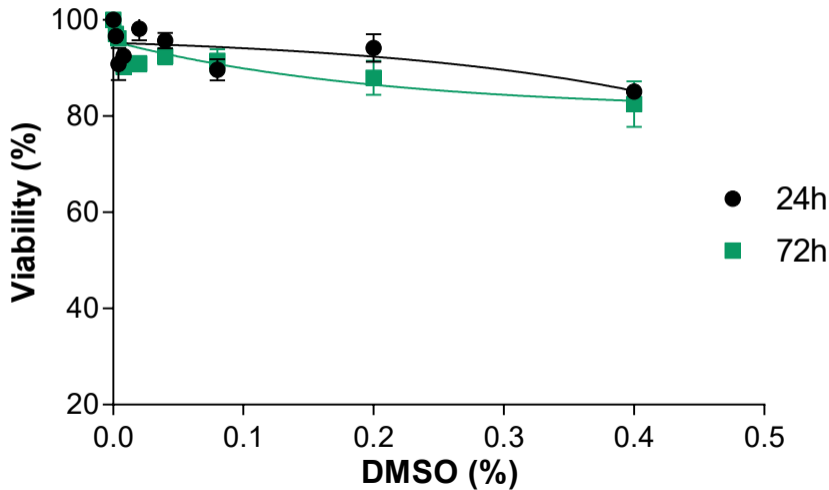

Supplement: Supplementary file 1 [file ijms-24-05055-s001.zip › Suppl_Fig_1.pdf]

# IL8

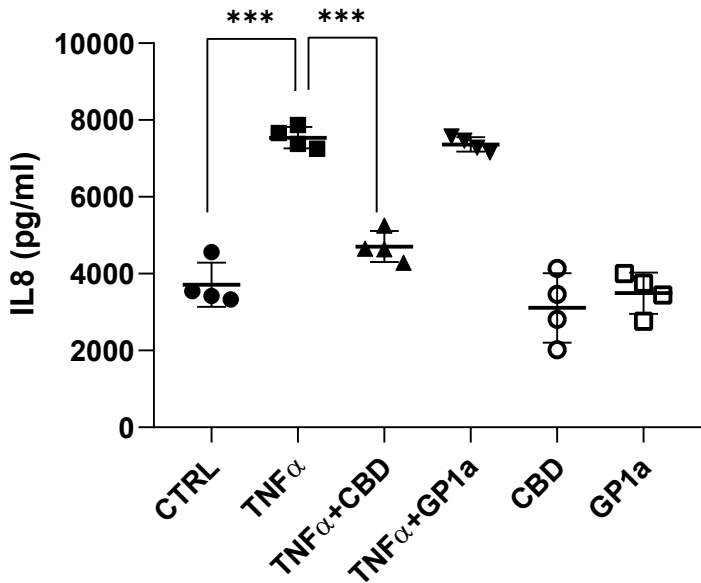

Supplement: Supplementary file 1 [file ijms-24-05055-s001.zip › Suppl_Fig_2.pdf]

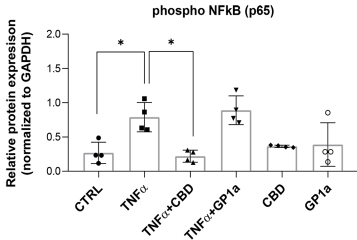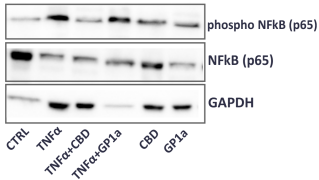

Supplement: Supplementary file 1 [file ijms-24-05055-s001.zip › Suppl_Fig_3.pdf]

## KEAP1

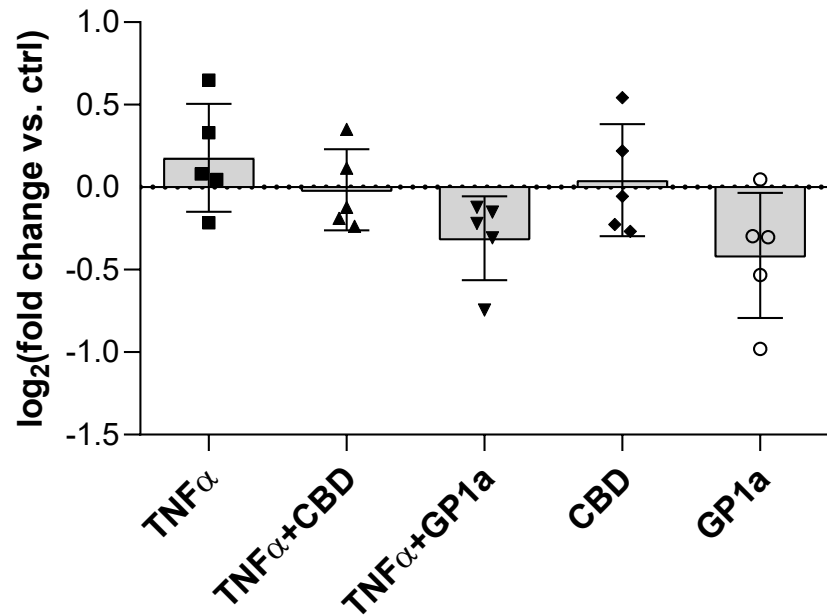

## NQO1

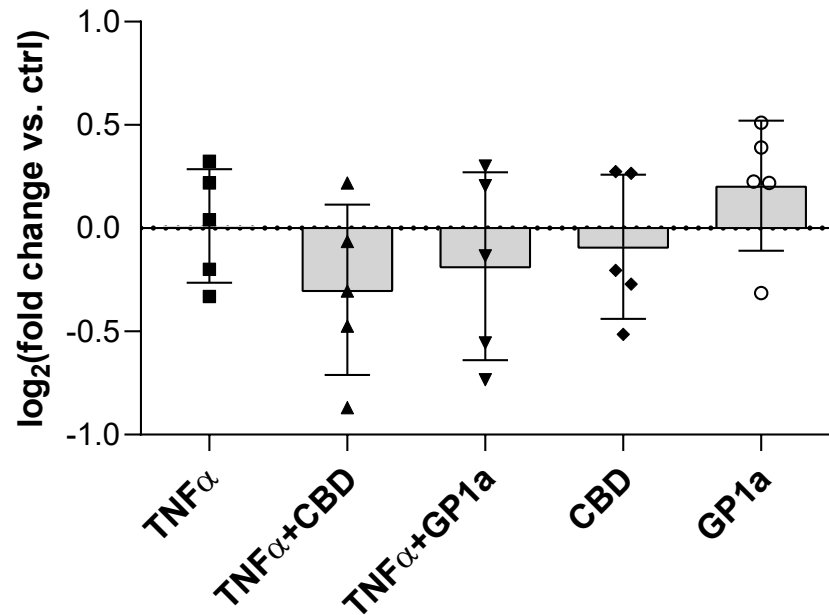

Supplement: Supplementary file 1 [file ijms-24-05055-s001.zip › Suppl_Fig_4.pdf]

# IL8

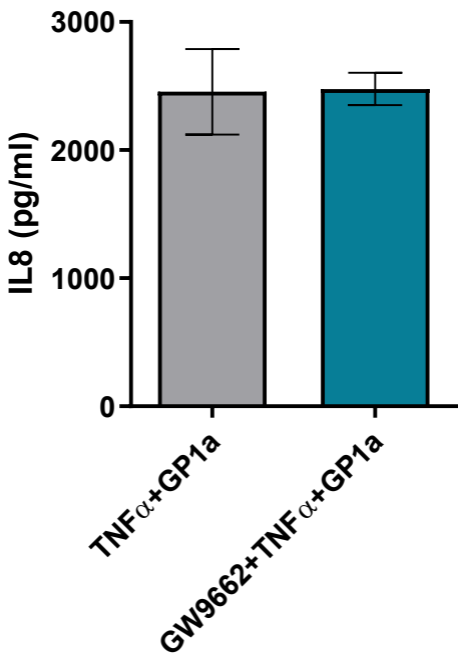

Supplement: Supplementary file 1 [file ijms-24-05055-s001.zip › Suppl_Fig_5.pdf]
